# Sensorimotor event: an approach to the dynamic, embodied, and embedded nature of sensorimotor cognition

**DOI:** 10.3389/fnhum.2013.00912

**Published:** 2014-01-03

**Authors:** Oscar Vilarroya

**Affiliations:** ^1^Departament de Psiquiatria i Medicina Legal, Universitat Autònoma de BarcelonaCerdanyla del Vallès, Barcelona, Spain; ^2^Fundació Institut Mar d’Investigacions MèdiquesBarcelona, Spain

**Keywords:** evolutionary psychology, extended cognition, situated cognition, active externalism, sensorimotor event, sensorimotor cognition

## Abstract

In this paper, I explore the notion of sensorimotor event as the building block of sensorimotor cognition. A sensorimotor event is presented here as a neurally controlled event that recruits those processes and elements that are necessary to address the demands of the situation in which the individual is involved. The notion of sensorimotor event is intended to subsume the dynamic, embodied, and embedded nature of sensorimotor cognition, in agreement with the *satisficing* and *bricoleur* approach to sensorimotor cognition presented elsewhere (Vilarroya, 2012). In particular, the notion of sensorimotor event encompasses those relevant neural processes, but also those bodily and environmental elements, that are necessary to deal with the situation in which the individual is involved. This continuum of neural processes as well as bodily and environmental elements can be characterized, and this characterization is considered the basis for the identification of the particular sensorimotor event. Among other consequences, the notion of sensorimotor event suggests a different approach to the classical account of sensory-input mapping onto a motor output. Instead of characterizing how a neural system responds to an external input, the idea defended here is to characterize how system-in-an-environment responds to its antecedent situation.

In a previous paper (Vilarroya, 2012), I introduced a set of neural processing principles and evolutionary constraints that should be taken into account in the characterization of sensorimotor cognition. I also reviewed evidence supporting the choice of the set of principles, and I assessed how such principles apply in two cases, object perception-action and peripersonal space. The aim was to emphasize the importance of focusing sensorimotor models on how evolution shapes functional paths to adaptations, as well as to adopt fitness maximization analyses of cognitive functions. The analysis yielded the view that neural systems should not be seen as a seat of optimal processes and circuits addressing particular problems in sensorimotor cognition, but as a set of *satisficing* and tinkered components, mostly not addressing the problems that are supposed to solve, but solving them as secondary effects of the engaged processes.

The aim of this paper is to provide an outline of the building block of sensorimotor cognition, which can be used to characterize this type of *satisficing* and *bricoleur *cognitive systems. The basic idea is that sensorimotor cognition must be understood as a coupling between the neural system and the external environment that deals with the specific situation the individual is experiencing. Hence, the logical outcome of such an approach is to establish the ongoing event, the particular situation in which an individual is involved, as the point of departure for the characterization of sensorimotor cognition.

The paper is organized as follows. I will first describe the neural processing principles that were presented elsewhere (Vilarroya, 2012). I will then introduce the notion of sensorimotor event as the way to adopt such neural principles in the characterization of sensorimotor cognition. The notions of affordance of the event and that of event’s functionality will be then described. In the next section I will focus on the embodied and embedded nature of sensorimotor events, and subsequently I develop how events are integrated in sequences and co-occur with other events. I will conclude with a discussion focused on how adopting the notion of sensorimotor event affects the classical framework of sensory-input mapping into a motor output.

## NEURAL PROCESSING PRINCIPLES

In a previous paper (Vilarroya, 2012) I showed that cognitive adaptations were obtained through a functional organization of neural systems that complies with a number of neural processing principles. The principles are the following:

### IN-FOCUS

The neural system selectively and contextually processes part of the all the available signals in order to manage the solicitation of the situation in which it is involved.

### AD HOC

Many studies are increasingly showing that the neural system is a much less modular than it had been previously thought (Anderson, 2010). In this sense, the position I defend is to assume a contingent level of functional specialization, where nervous systems can be seen to have specialized circuits, because some of its elements (e.g., sensors) are contingently constrained to process certain data (e.g., electromagnetic waves) and because some circuits undertake some types of specific tasks (e.g., visual), devoting some fixed resources to them.

### TRANSVERSAL

The interaction among different sensorimotor modalities is pervasive for all neural processes.

### HETERARCHICAL

The nervous system processes signals at different stages, though these stages do not imply strict boundaries, nor sequential processing; namely, there are no strict boundaries between sensory, perceptual, and motor stages, nor is there a strict bottom-up or top-down hierarchy.

### MODULATED

The neural system is endowed with specific mechanisms that regulate the processing of neural signals as a function of the endogenous relevance of such data. Such modulatory functions have the property of biasing, controlling, and modifying neural processing endogenously, through reward/punishment, inhibition/activation, and other similar constraints.

### OPEN

An open system is a system that is necessarily in interaction with an environment. The brain has also been considered as an open system in which information is exchanged with the environment. However, as I understand it, the open principle also applies to the functional characterization of the system. Hence, the idea is that the brain is an open system whose sensorimotor capabilities must be characterized taking into account the brain and the environment. There is an interchange of information between the brain and the environment, but also, functionally, it is necessary to characterize the neural system and the environment as a functional continuity. The environment has an active role in the cognitive activity of the system, and it is necessary to characterize and model the functionality of the system.

## SENSORIMOTOR EVENT

Elsewhere, I have (Vilarroya, 2012) assessed the suitability of this set of principles in two cases: object perception, and peripersonal space. As mentioned before, the picture we get from applying such a set of principles suggests that sensorimotor cognition must be understood as a coupling between the neural system and the external environment, and such a coupling dynamically addresses the ongoing situation. This should come as no surprise. In a dynamic context, the minimal function of the neural system is to help the individual to get from the present moment into the following one by satisfying the system’s needs, nothing more than this. What happens in the neural system within the framework of a concrete situation is what we need to understand about the brain to characterize it. We could in fact understand the function of the nervous system to characterize the present situation, according to the needs of the system (what happens now that it is relevant to me), and act upon it selecting the most appropriate action, according to the needs of the system (how do I get what I want?).

Accordingly, the logical outcome of the approach defended here is to establish the ongoing event, the particular situation in which an individual is involved in, as the point of departure for the characterization of sensorimotor cognition. In the rest of this paper I will try to outline the main features of the notion of sensorimotor event. Let me first provide a working definition of sensorimotor event:

### SENSORIMOTOR EVENT

A sensorimotor event is a neurally controlled event during which its affordances are addressed by the engagement of a number of functionalities.

By event I understand a particular temporally bounded situation, and by neurally controlled I assume that the event’s duration, processing and register is managed by the neural system. These are two uncontroversial notions, and thus I will not address them in detail. Instead, I will now explore the notions of affordances and functionalities, which require further explanation.

### EVENT AFFORDANCES

If we see a glass of water on a table, and we realize that we are thirsty, then the open ended number of possible sensorimotor actions available to the individual will be constrained to a number of sensorimotor responses that are adapted to the situation. The fact is that every sensorimotor event is preceded by another event that primes an adaptive response. Such a response is constrained by neurobiological predispositions (selected for by evolution) and previous experiences of the individual. The set of constrained responses that are primed by the antecedent event will be defined here as the *affordances* of the event.

I borrow this notion of affordance from the domain of ecological psychology (Gibson, 1986). Originally, the notion of affordance referred to a property of an object or of the environment that offers a biological organism the possibility of an action; an affordance would be a precondition for a motor activity (Greeno, 1994). Typical affordances include objects that can be manipulated in a certain way, such as a mug’s handle affords holding it, or substances afford being eaten or drunk. Affordances, once detected, have adaptive value for the organism that detects them.

The properties of the affordances were traditionally attributed strictly to the object or the environment. Now it is recognized that the affordance cannot be simply in the stimulus or in the environment. Affordances are now conceived as relations between the features of a situation and the abilities of an individual (Chemero, 2003). The notion of affordance has been in fact revisited and re-defined in a sense that better fits my proposal here. An affordance is defined now as a relational property that emerges from matching the perceived physical features of an object (e.g., size, shape, texture, density) and the agent’s abilities and requirements. More specifically, Ellis and Tucker (2000) introduced the notion of “microaffordance” to indicate the activation of sensorimotor components (reaching and grasping processes, such as establishing the size and distance from an object) suitable for interacting with specific objects and adapted to the individual needs. A particular microaffordance, such as the judgment of distance with respect to a given object, would vary according to the action intentions of the individual (Witt et al., 2004).

In my opinion, the notion of affordance in which the environment “primes some constrained response by the organism” can be likened to the way an antecedent event “primes” a response from the ensuing event. Basically, I understand an event’s affordance as the constrained responses that a previous event primes the ensuing event.

The event’s affordances are the critical element of an event because the neural system will prioritize the management of the affordances at issue; affordances guide, define, bias the activity of the system. Furthermore, affordances determine the temporal limits of the event: an event ends when the affordances are effectively addressed, or abandoned due to inability to deal with them (a mug being too far away) or to particular contingencies of the situation (a phone rings while trying to grasp a mug).

### EVENT’S FUNCTIONALITIES

Consider what happens when we perform the previously simple act of reaching out and picking up a glass of water. After *identifying* the glass among all the other possible objects on the table, we begin to *reach out* with our trunk, body, and hand toward the glass, *choosing a trajectory* that *avoids* the lamp which is in the middle of the path toward the glass. At the same time, our fingers begin to *conform to the shape and texture* of the glass well before we make contact with it. As our fingers *turn around* the glass, the initial forces we generate to lift the glass *are finely tuned to its anticipated *weight – and to our *predictions* *about the friction coefficients and compliance of the material* from which the glass is made.

The event of seeing a glass of water and recognizing our thirst primes a set of affordances that are addressed by a number of *functionalities*. By functionality of a sensorimotor event I understand a particular operation carried out by the system that deals with the event’s affordances. It is important to note that the notion of functionality is a theoretical construct. It is the outcome of a functional analysis of the event (Cummins, 1985), and thus it depends of the way the analysis characterizes the functional elements and the interaction among them. Such an analysis can have alternatives which can provide equivalent characterizations of the event. However, a functionality intends to describe a specific continuum of neural, bodily, and environment processes and elements articulated in a determinate way. All the components in the continuum play an active causal role, and together they account for the organism–environment behavior.

Functionalities usually correspond to the sensorimotor abilities that a system shows, such as, for example, the different skills that are deployed to perceive depth: motion parallax, motion depth, or stereopsis. Likewise, as I have indicated in the example above, grasping a glass of water involves, among other functionalities, reaching out a hand toward an object, estimating the object’s size, orientation, and position with respect to the hand, calibrating the grip according to the size, weight, and material of the object, etc. A functionality can nevertheless be used to describe a high-level functional description, such as “depth perception” or “postural control,” and at the same time it can be broken down into simpler functionalities that account for the high-level functional description. Hence, depth perception can be analyzed into motion parallax, depth motion functionalities, and postural control in anticipatory postural adjustments, anticipatory synergy adjustments, among other possible functionalities (Klous et al., 2011).

The particular functionality selected in an event is determined by the affordance at issue among all the available functionalities in the system. Note that it is the situation, not simply the elements present in the situation, what will determine the functionality in question. A situation where the object is perceived without intention to act on it will recruit different functionalities than a situation in which we intend to act on it. For instance, Ganel and Goodale (2003) found that when people were asked to make perceptual judgments about the width of different rectangular-shaped objects, their judgments were always affected by differences in the length of those objects. In other words, vision-for-perception always took into account the overall shape of the objects. But when the same subjects were asked to pick up the objects, they responded differently: their grasping movements were unaffected by the differences in the non-relevant dimension of the objects. In other words, vision-for-action focused on only the most relevant dimension of the goal object (in this case its width) without being influenced by its length. An interesting observation by Ganel and Goodale (2003) is that when subjects were asked to pantomime the grasping movements without actually touching the target objects, their grasping was then affected by the differences in the length of the object, just as it was in the perceptual judgment task. This presumably reflects the fact that the production of a pantomimed movement utilizes perceptual rather than direct visuomotor processing.

Finally, it is relevant to point out that a functionality does not assume specific claims about how to be instantiated, nor does it require that there be a specific set of processes, networks, or systems dedicated to deal with the functionality at issue. In agreement with the *bricoleur *and *satisficing *constraints (Vilarroya, 2012), a functionality can in fact be the outcome of a specific sub-system dedicated to it, or the outcome of a sub-system or set of sub-systems not focused on such a functionality, but efficient in dealing with it.

## EVENTS ARE EMBODIED AND EMBEDDED

The proposal that I present here conceives events as sensorimotor units that are embodied and embedded, in agreement with the neural processing principles presented above and elsewhere (Vilarroya, 2012). Therefore, the characterization of an event should take into account the bodily and environmental elements that are essential in the management of the affordances at issue. If event_*n*_ comprehends a continuum of sensorimotor neural processes, as well as bodily and environmental elements, event_*n*__+__1_ will comprehend another continuum of neural, bodily, and environmental elements that deals with the affordances that event_*n*_ raises. This continuum of processes and elements can be characterized, and this characterization should be the basis for the identification of an event.

There is now a tradition on which the proposal presented here is partially based, known as the “extended mind” or “active externalism” approach (see, for example, Menary, 2010). The agendas and particular hypotheses of extended mind approaches differ among them and also from mine^1^. In general, the basic idea behind extended-mind approaches stems from the observation that, when satisfying some tasks, a part of the world/environment functions as an element that complements those neural processes carried out in the brain and which are necessary to fulfill a given cognitive task. The notion of extension or scaffolding includes different kinds of external aids and support. The examples go from the aid of pen and paper to execute arithmetical operations, up to the very use of language to derive, for example, arguments. The rationale is that in many cases the human organism is linked with an external entity in interaction, creating a “coupled” system that can be seen as a cognitive unit in its own right. All the components in such a system play an active causal role, and together they govern behavior in the same sort of way that cognition usually does. If we remove the external component, the system’s behavioral competence declines, just as it would if we removed part of its brain. The hypothesis is that this sort of coupled process counts equally well as a cognitive process and system, whether or not it is wholly in the neural system. Hence, among other things, such processes require that the characterization of the neural system be extended beyond the limits of the brain and the body to include elements of the environment. Let me illustrate how active externalism can be applied to sensorimotor cognition.

### LOCOMOTION

As it has been progressively confirmed in sensorimotor literature, neural circuits implicated in locomotion interact continuously with the environment. Locomotion based purely feed-forward commands is insufficient for animals to deal with changing contingencies in the environment, because of the unpredictable nature of environmental elements. Indeed, the emerging picture of locomotion in natural habitats is a series of temporally varying and interactively complex events, rather than constant and straight locomotion program. Flying insects provide a good example of how this coupling between body mechanics and the external environment can generate complex event sequences. Insect wings must flip over at the end of each wingbeat as the wing reverses direction. The lift force depends sensitively on the timing of the flip (Sane and Dickinson, 2002), and the rotation is driven by passive interactions with the air (Bergou et al., 2007) showing the role of the environmental interactions in defining motor output.

However, recent theoretical approaches to locomotion do not only consider that continuous feed-back is necessary, but that locomotion cannot be characterized, and modeled, without assuming a continuum of activities that involve the nervous system, the biomechanics of the body, and the environment (Chiel et al., 2009). As Smith (2005, p. 286) puts it, “the ability that makes alternating leg movements is not strictly in the brain, not the body, nor the world but in the interaction of a particularly structured body in a particularly structured environment.” Therefore, these new models assume that bodily and environmental elements also need to be integrated in locomotion models (Chiel et al., 2009).

### GRASPING

Another illustrative domain of the embodied and embedded nature of events is object grasping. Successful grasping requires a series of processes that analyze an object’s dimensions and its surroundings while selecting the appropriate movement path and hand configuration. Information about intrinsic object features, such as absolute size, shape, and color of the object, as well as extrinsic information, such as distance and orientation in the environment, need to be processed in order to carry out an efficient movement. For example, grasping requires using more fingers if the object is perceived as heavy or slippery, or it prescribes executing the movement more slowly if the object is fragile (Hoff and Arbib, 1993; Fikes et al., 1994).

Classic feed-forward models held that grasping motor actions were defined before onset of the movement and that feedback loops were engaged toward the end of the movement trajectory, if at all. However, such goal-directed movements are not always accurate and object and environmental properties may change before the movement is complete; thus, sensorimotor information may also be needed after movement onset and guide necessary corrections (Hoff and Arbib, 1993). Extant studies (Karok and Newport, 2010) recognize that neural motor commands in grasping are crude and not precise, and that environmental and biomechanical elements continuously change during movement. Therefore, now the contention is that continuous feed-back is necessary after movement onset to apply the necessary corrections. Indeed, feedback plays an important role in acquiring and developing motor skills, and this particularly applies to grasping movements (Schenk et al., 2004). Object location, for example, cannot be programmed before movement onset, and it must be continuously updated, while it is externally maintained by the object itself. The fact is that, until recently, the widely held hypothesis was that the location of an object was coded in gaze-centered coordinates, which was adjusted in conjunction with eye movements. Now, the favored hypothesis is that object grasping depends on current eye position, suggesting that the object properties are continuously processed after each saccade, and therefore that grasping depends of this gaze-centered updating stage (Selen and Medendorp, 2011). In sum, the environment must not only be the subject of a continuous feed-back, but it also plays a role in itself in the performance of grasping actions.

Special environments are also an interesting way to understand the sensitivity of grasping to environmental contexts. One recent study focused on sensorimotor deficits in single and dual visuomotor tasks during spaceflight (Bock et al., 2010). The authors explored the performance of astronauts in pre-flight and in-flight, after months at the International Space Station (ISS). The study showed that even after prolonged exposure to the space environment, subjects’ visuomotor performance remained compromised in both types of tasks, although performance returned to preflight levels within days of returning to earth. The deficits could not be explained by any cognitive deficit of the individuals, and thus were attributed to the role of the context. In other words, cognitive performance of individuals seems to be sensitive to all the elements that comprehend the individual-in-an-environment.

Pathological conditions of object grasping also illustrate the critical role of the environment. Brain damage caused by stroke can lead to apraxia, an impairment frequently involving defective motor actions grasping or tool using. A usual paradigm to study the ability of apraxic patients involves three different execution modes: pantomime use of a tool, its demonstration, and the actual use of a tool. In general, it is reported that patients with apraxia show more salient deficits in the pantomime of tool use than during actual use (Randerath et al., 2011). However, this result cannot be explained in terms of classical deficits of neurological conditions. According to the “severity hypothesis,” there is a continuum of task-difficulty: actual use is the most difficult task, because the patient has to move a hand with a tool, and pantomime is the easiest, because the patient must only move a hand. Yet, neurological patients showed the inverse effect in their study: they were better at the actual use of the tool with the hand. The authors explain this paradox by assuming that in the actual use of the tool the environment, i.e., the tool, provides resources, and information that are absent in pantomime. The tool constrains the possibilities of an action by reducing the degrees of freedom for the required action, and therefore it makes it easier.

Finally, computational models of object grasping have begun to integrate the environment in their formalization. In principle, algorithms for controlling complex bodies in grasping activities were supposed to represent the bodily and environmental elements. However, an alternative view is that the mechanics of the moving parts interaction with the environment may actually simplify control (Nishikawa et al., 2007). These new models include parameters that are environmentally maintained: movement planning is assumed to rely on accessing the extrinsic properties of an object (e.g., its location, orientation, etc.), as well as the intrinsic properties of the object (e.g., shape, size), without internally coding for them. In sum, the environment is beginning to be included in computational models as an intrinsic part in the computations of grasping movements.

### DEXTEROUS SKILLS

Extant studies on sequence learning of sensorimotor skills show the critical role of the bodily and environmental elements. Take the example of Crump and Logan (2010a,b) who have shown that skilled sequences, such as typewriting, are extremely sensitive to the particular environment involved in the skill. They investigated the role of physical properties of the keyboard in typing, specifically focusing on tactile, haptic, and proprioceptive features. In their study, typists carried out single-word and paragraph typing tasks on a three type of keyboards: a regular keyboard, a laser-projection keyboard, and a deconstructed keyboard, made by removing successive layers of a regular keyboard. The response times for the keystrokes, the interval between keystrokes, and the error rate increased significantly in the non-regular keyboards, even if the three types of keyboards required the same visuomotor skills from the typists. In other words, the performance of the typists can only be characterized taking into account the actual environment, rather than analyzing the visuomotor task to accomplish in isolation.

To sum up, recent sensorimotor literature provides abundant evidence in support for the assumption that sensorimotor events are embodied and embedded. In this sense, the idea suggested here is that the characterization of sensorimotor events should *always* include those neural processes that deal with the affordances at issue, but also those bodily and environmental elements that are *essential* to account for the individual’s behavior.

## EVENTS BELONG TO SEQUENCES AND CO-OCCUR WITH OTHER EVENTS

Sensorimotor events have temporal boundaries, with beginnings and endings, even though what constitutes a boundary is not yet well-established, but it is directly related to the affordance of the event: what the situation demands, and when it is satisfied or discarded. It is important to note, though, that these boundaries are to a certain extent conventions of the characterization, in the sense that there are no discontinuities in processing, and that many times an event overlaps with the following one. For instance, in dexterous typing, high-speed films of typists typewriting show that finger movements often occur in parallel, and the finger movement for one keystroke often begins before the finger movement for the preceding keystroke ends (Flanders and Soechting, 1992).

### EVENTS IN SEQUENCES

Sensorimotor cognition is intrinsically dynamic, and it usually comprehends sequences of events. Events are indeed seldom temporally autonomous; they usually belong to a sequence. The fact is that the ability to pattern and register sequence of events is fundamental to neural systems. Sequences are pervasive in the whole neural system, from the most basic neurochemical processes, up to the most high-level cognitive functions. A wide variety of skills such as comprehending and producing language, type-writing, or playing musical instruments rest on the ability to process structured sequences of events (e.g., speech sounds, movements) and to assemble elementary responses into novel action sequences (e.g., key-presses on the keyboard, phonemes when listening speech). Furthermore, sequential knowledge enables organisms to predict what will happen next, where it will happen, and how to react to it, and thus constitutes the basis for the anticipatory control of sensorimotor cognition.

Sequences can be of four different types: (a) pre-established and fixed, such as reflexes; (b) learned, and strongly routinized, such as the dexterous car driving, typing, walking, etc.; (c) learned and open, that is, not fixed, although codified, such as speech; (d) primed by experience and loosely predictable, such as exploring the environment. All sensorimotor events in principle belong to at least one of these four types; it is extremely unusual that an event does not take part in a sequence.

There is ample evidence on the role of sequences in sensorimotor cognition (Kurby and Zacks, 2008), and a recent study (Pfeiffer and Foster, 2013) has shown how an event-based approach to sequences can be traced down to a group of hippocampal cells that predict future sensorimotor events. The extant literature on the role of events in sensorimotor cognition has nevertheless focused on the discreteness or continuousness of perception and/or motor behaviors (VanRullen and Koch, 2003; Siegel et al., 2012). In contrast, studies on sequences have not addressed the nature of events as particular elements in themselves; rather, they have generally considered events as simple steps in a sequence.

Here I would like to emphasize the relevance of focusing on the events in sequences as *singular *elements. Events can belong to a specific sequence, and as such, they can be registered in memory as part of the sequence, but they are also registered as elements with particular properties. Therefore, they contain information about their belonging to a specific sequence, but they also have properties that are *not* related to being part of a sequence. Hence, it would be wrong to characterize events strictly as the automatic steps in the sequence. Sensorimotor events are singular elements that belong to sequences but have specific properties of their own. Take the example of postural control. Postural control involves managing sensorimotor cues related to whole-body position and motion in space and to the displacements of the environment relative to the individual. Such a management is extremely dynamic and requires an efficient integration of all sensorimotor cues, so that each event in a postural sequence has a critical importance. For instance, orientation information from various sensory modalities can be abruptly unavailable (for instance, when lights go off) or can become inaccurate (when support surfaces are instable). Hence, postural control mechanisms must be adjusted to maintain stance at each stage. Studies have shown that postural control manages these changes through dynamic reweighting of sensorimotor cues in an eventual fashion. Dynamic reweighting manages the modifications required to maintain postural control, by proportionally adjusting the different sensorimotor cues in an event by event basis; unreliable information is down-weighted, then gradually up-weighted when it becomes valid again in ulterior events (Peterka, 2002). In other situations, such as maintaining balance on a level fixed surface with eyes closed, postural control uses primarily proprioceptive sensorimotor cues, which signal body motion relative to the feet, but if the surface begins moving, ulterior events discount the proprioceptive information and shift toward increased reliance on detecting body orientation with respect to the vertical (Peterka and Loughlin, 2004). In sum, sensorimotor sequences rely on events as the building blocks of an efficient behavior.

### CONCURRENT EVENTS

At a given moment, an organism might be managing different sensorimotor events at the same time. In this sense, sensorimotor events may be concurrent with as many other sensorimotor events that are necessary to deal with the affordances of a particular situation. For example, an individual might be dealing with balance events, and at the same time trying to grasp a ball in the air. Concurrency of events is uncontroversial. All models of sensorimotor cognition acknowledge explicitly or implicitly the concurrency of different sensorimotor processes. However, the differences between models lay in the autonomy of concurrent processes. This has been usually addressed in discussions about the modularity of sensorimotor processes, even if the question of temporal concurrency has not been a main vector of such discussions. I have stated my position on modularity elsewhere (Vilarroya, 2012). As I indicated above, I assume a contingent level of functional specialization, where nervous systems process special tasks, because sensors are physically constrained to process certain data and because some circuits repeatedly carry out certain types of specific tasks, devoting some fixed resources to them. The fact of the matter is that the degree of autonomy of sensorimotor events depends on the contingencies of the situation and/or the task at hand. Concurrent events are open to modulation and interconnection among them if the situation or the task requires it. Let me demonstrate this idea with some examples.

Dexterous skill execution is a domain where variable modularity has been shown. Logan and Crump (2009,2011) have studied the interaction between sensorimotor domains in various skills, such as in typewriting and musical execution. Typewriting seems to be strongly modular and informationally encapsulated (but see Jasmin and Casasanto, 2012). On the one hand, typewriting involves a set of processes that transform words into keystrokes, as well as control the motor execution of fingers and hands; on the other, typewriting involves a set of processes that link the selection of words to type with the motor execution processes. Logan and Crump (2009,2011) suggest that these two modules are informationally encapsulated. Typewriting seems to be exclusively focused on the result of performance. The outcome of typing is a string of letters correctly typed. In contrast, musical execution is an example of a much less modular architecture between sensorimotor domains. Playing music is different in that the musical performance itself matters more than the exact execution of the notes. The musical expressive dimension is a product of refinements of the interaction between the motor execution and the emotional domain. Guitar players use note sliding, timing, and vibrato to convey emotion. Piano players handle emotion with timing and the strength of key striking. In sum, even if both typewriting and musical execution use similar sensorimotor processes, their modularities are different, as Logan and Crump (2011) recognize.

Another feature of the concurrency of events is that concurrent events can have a varying degree of autonomy, and at the same time they can affect each other in a variety of ways. For example, Novembre and Keller (2011) hypothesized that, if a musical context can induce specific expectations in the auditory domain (i.e., a specific tone), then the motor domain of such sequential learning should generate comparable expectations (i.e., the specific movement required to produce the expected tone(s)). In agreement with previous evidence for the audio-motor coupling in musicians (Drost et al., 2005), they showed that for experienced performers violations of syntactic rules in the musical domain also induce violations of syntactic rules in the motor domain. In a similar study, Drost et al. (2007) focused on how auditory-motor couplings are contextualized to their own instrument category in expert musicians. They used a task where musicians played chords on their instrument in response to visual stimuli, while they were presented task-irrelevant auditory distractors (congruent or incongruent) in varying instrument timbre. In such a task, they showed that pianists exhibited an interference effect only with timbres of their own instrument category. In a previous work (Drost et al., 2005), they required expert pianists to play chords on a keyboard in response to specific visual stimuli, while at the same time, task-irrelevant auditory stimuli (that could induce alternative incongruent motor actions) were presented. They found evidence that expert pianists were sensitive to incongruences and affected the motor response of the sequence. It therefore appears that concurrency of events is a common situation that has varying degrees of autonomy and interaction.

## DISCUSSION

The proposal presented here has been to establish the ongoing event, the particular situation in which a system-in-an-environment is involved, as the point of departure for the characterization of sensorimotor cognition. The sensorimotor event has been defined as a neurally controlled event that integrates all neural processes as well as all bodily and environmental elements that take part in addressing the affordances of the event at issue. The notion of sensorimotor event has been used to develop the basic idea that sensorimotor cognition can be better characterized as the response of an embedded and embodied system to a particular situation. Such a response depends on the set of constraints that the antecedent event poses to the ongoing event.

The content of the proposal presented here cannot be considered completely novel. There have been various attempts to characterize the notion of sensorimotor events (VanRullen and Koch, 2003; Kurby and Zacks, 2008). Moreover, the approach presented here can be related to long tradition that assumes an embedded and embodied view of cognition. In recent years, the embedded and embodied dimension has been accommodated in different ways by various approaches, such as that of ecological psychology (Gibson, 1986), sensorimotor contingencies (O’Regan and Noe, 2001), event-code theory (Hommel et al., 2001), and the approaches of Hurley (2001) or Smith (2005). A thorough analysis of how my proposal could be contextualized in these (and other) theories cannot be attempted here. Some critical differences can nevertheless be noted. For example, event-code theory presents a detailed theory about a common distal (abstract) code for perception and action, which cannot have a place in my proposal because I do not support an abstract representation of events. Furthermore, in contrast to my proposal, event-code theory does not give the environment a particular role to play. In the case of ecological psychology, I defend, in contrast to ecological psychology, that that there is relevant and irreducible internal neural processing in sensorimotor cognition. Sensorimotor contingencies theory, in its turn, is focused on understanding consciousness, which is not my aim here. Likewise, Linda Smith’s focus on learning cognitive capacities, and Susan Hurley detailed representational theory of cognitive competences are neither of them my objective here.

In my opinion, the view that I advance subsumes the findings that conceive sensorimotor cognition as a dynamic, embodied, and embedded sequence of events. Furthermore, the notion of sensorimotor event as presented here suggests, in my opinion, a particular consequence that is interesting to explore. In adopting the embodied and embedded sensorimotor event as the building block of sensorimotor cognition, I focus sensorimotor cognition on how an organism-in-an-environment responds to a *situation*, rather than on how a neural system responds to an *external input*. In other words, the classical framework of external input, internal processing, and external output can be turned around into a extended (neural, bodily, and environmental) antecedent, as input, and an extended consequent, as output.

**FIGURE 1:**
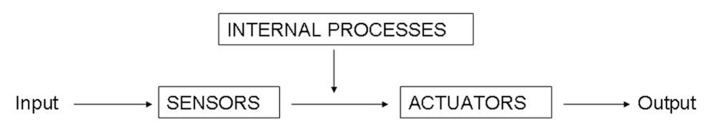
**The classical sensory input mapping into a motor output**.

**FIGURE 2:**
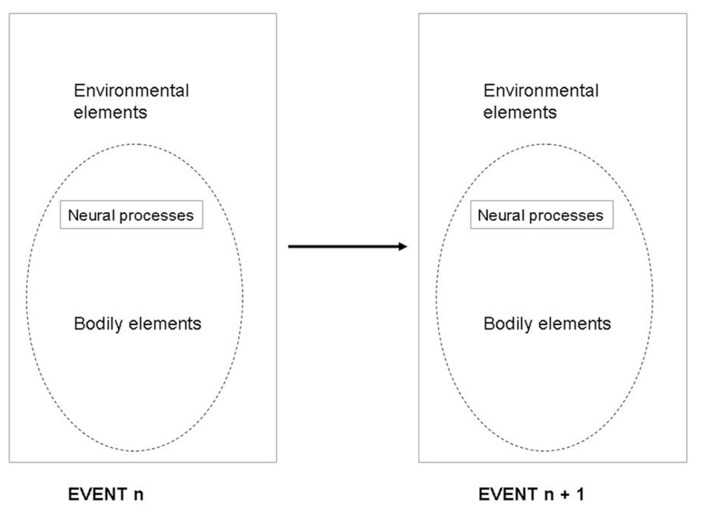
**The framework characterizing event_*n*_ mapping onto event _*n* + 1>_**.

Classically, the task of the nervous system was described as a function which maps an *n*-dimensional input space (from the sensors) to an *m*-dimensional output space (the actuators). This framework suggested that the mapping function uses a set of computations which transforms sensory data into motor data. Take the identification of a car coming toward you. In this situation, the classical framework states that the sensors receive visual information that is processed into the category “car” object, with the property of moving toward you at high speed. Then, this information is processed and transferred into a motor action associated with something like “jump aside.” However, the proposal presented here suggests a modification in this scheme. Instead of assuming the classical sensory input mapping into a motor output (**Figure 1**), it implies an event_*n*_ mapping onto event_*n* + 1_ (**Figure 2**). In this framework, the input would not be a sensory pattern, but an integrated sensorimotor pattern coupled with the relevant bodily and environmental elements, and the output would also be an integrated sensorimotor pattern coupled with the relevant bodily and environmental elements. In the situation of the approaching car, the proposal presented here suggests that we treat each stage as a *response* to the previous one. The event_*n*_ provides a set of affordances that will cause event_*n* + 1_ to begin motor responses of “jumping aside,” even before the concept of “car” is active, constrained by neurobiological bias and memory of previous similar experienced events. Indeed, the first sensory information received from the car is processed in an event containing certain sensorimotor particularities that primes a new event with motor actions that could be associated with initiating an avoiding behavior. This new event processes new sensory information integrated with motor information and the relevant environment (e.g., standing on a stable or unstable support) that primes the following event. The idea is that this scheme is reproduced in all sensorimotor events.

In my opinion, this framework provides a tool to characterize the dynamic, embodied and embedded nature of sensorimotor cognition. For example, in the case of the approaching car, the sensory information of the car first processed in the event could be undistinguishable from the information coming from speck of dust crossing the field of view of the individual. Thus, the event responding to both situations could be alike. However, in the subsequent event, the sensorimotor information will produce two different sensorimotor scenarios. The different dynamics of the interaction between the sensory and motor processes in the two situations will create divergent sequences sensorimotor of events for each situation. For one thing, the sensorimotor information produced by the relation between the body position and the speck of dust will be different than the sensorimotor information produced by the relation between the body position and the car, much before the concepts of “car” or “speck of dust” would be active, and even before the sensory data produced by the speck of dust and by the car could be distinguished in sensory terms alone.

In sum, I argue that the sensorimotor-event framework is better suited to account for the way sensorimotor information is processed in the neural system. In any event, this is an empirical hypothesis, and thus it is subject to testing and evaluation. In my opinion, there are a variety of predictions that can be envisaged, which for brevity of exposition, I will summarize in the following conditions:

*Environmental condition*. The sensorimotor event is based on the idea certain environmental information need not be represented in the neural processing, because it is ready available and it plays an irreducible role in the sensorimotor competence of the individual. This has the consequence that, among other things, changing critical environmental properties during an event will change the ongoing sensorimotor functionalities. Indeed, if the critical environmental properties are manipulated, the efficient processing of any task that the neural system is carrying out will be distorted. The study of sensorimotor performance in spatial contexts (Bock et al., 2010) is an example of how this condition can be studied (which can be set in virtual-reality contexts).

*Embodiment condition*. Embodied features are a constitutive part of sensorimotor events. Hence, the modification of such features will compromise the properties of the sensorimotor event, in the same way that in the environmental condition. As an example, the study of Randerath et al. (2011), in which patients with apraxia show more salient deficits in the pantomime of tool use than during actual use, can be a good illustration of how to test the proposal presented here. Likewise, a recent study Warburton et al. (2013) show how to probe the connection between the event and the embodiment of cognition.

*Processing condition*. An event is a neurally controlled element, and thus subject to a number of processing constraints. For example, events have boundaries, and such boundaries have processing properties that can be probed. Among other things, event boundaries are associated with changes in processing time, space, and goals (Kurby and Zacks, 2008) that should be reflected in the embodied and embedded properties of the event.

*Developmental condition*. Sensorimotor cognition and its integration within events have a developmental dimension. Hence, it would be reasonable to think that the development of sensorimotor-events’ properties such as, for example, event segmentation, will correlate with the development of sensorimotor competence.

*Pathological condition*. In contrast with the developmental dimension, we have the pathological dimension. Indeed, sensorimotor events are subject to loss of its processing capacities, and thus, one can infer that there will be a correlation between sensorimotor deficits and loss of event’s properties (Kurby and Zacks, 2008).

Another set of tests can be set in the interaction of the previous conditions. In sum, even if other conditions and predictions could be added, those included illustrate the sort of predictions and tests that can be used to probe the hypothesis presented here. It is important to note, though, that such a proposal is incomplete; it provides a basis for characterizing the building block of sensorimotor cognition but, in order to count as an efficient framework, there are many additional developments that must be addressed. Among other things, the proposal must be further developed to account for how different sensorimotor processes are integrated within a single event, how the elements of an event, such as its boundaries, are processed, how the processes belonging to an event are registered in memory, and how such processes are then recruited and applied in future situations, as well as many other critical issues. Additionally, it will also be necessary to provide a formalization of the extended event framework. A critical challenge in this characterization is that the relevant environment should be included in the components of an event. As has been mentioned before, there are already computational models that use the environment as part of the computational process. However, the bodily and environmental contributions have not been generally formalized in models of sensorimotor cognition. All of these issues, and many more that will be forthcoming, should be the content of an further work.

## CONCLUSION

The notion of sensorimotor event suggests a different approach to the classical account of a sensory-input mapping onto a motor-output. Instead of characterizing how a neural system responds to an external input, the idea defended here is to characterize how a system-in-an-environment responds to its antecedent situation. Applying and developing this notion as the building block of sensorimotor cognition will hopefully account for its dynamic, embodied, and embedded nature.

## Conflict of Interest Statement

The author declares that the research was conducted in the absence of any commercial or financial relationships that could be construed as a potential conflict of interest.

## Footnotes

1 The extended mind approach has indeed raised a heated debate about the characterization of cognitive and mental processes (see for example the issue 28, 2009 of Topoi). These discussions have focused on the constitutional and causal arguments of what can be considered mental, and thus, of the ontic status of the external elements in the characterization of the extended processes. In contrast, the proposal I defend here concerns the functional analysis of the coupled system brain environment that accounts for sensorimotor cognition. I assume here the stance that events are extended, without addressing if such extended processes and elements should be considered mental or not. Moreover, I defend that all sensorimotor processes must be considered to be embodied and embedded, at least from a functional point of view, which is not generally accepted by all active externalism approaches.
